# Delirium-directed interventions and long-term cognitive outcomes in critically ill adults: a systematic review of randomized clinical trials

**DOI:** 10.3389/fneur.2026.1833028

**Published:** 2026-05-29

**Authors:** Shradha Pandurang Kakde, Niraj Arora, Meghnath Kakde, Shubhangi Kakade

**Affiliations:** 1 MGM Medical College and Hospital, Chhatrapati Sambhaji Nagar, Maharashtra, India; 2 Department of Neurology, University of Missouri, Columbia, MO, United States; 3 Smt. Kashibai Navale Medical College and Hospital, Pune, Maharashtra, India

**Keywords:** cognitive impairment, critical illness, ICU delirium, post-intensive care syndrome, randomized clinical trials, rehabilitation

## Abstract

**Background:**

Delirium is a common manifestation of acute brain dysfunction during critical illness, strongly associated with persistent cognitive impairment among ICU survivors. Although delirium duration and severity have been linked to adverse neurocognitive outcomes, it remains uncertain whether delirium-directed interventions implemented during ICU admission or after discharge can meaningfully modify cognitive trajectories. We conducted a systematic review of randomized clinical trials to evaluate whether delirium-directed interventions improve long-term cognitive outcomes in adult ICU survivors.

**Methods:**

This PRISMA-compliant systematic review was prospectively registered in PROSPERO (CRD420261295266). We included randomized clinical trials enrolling adults admitted to medical or surgical ICUs with acute respiratory failure requiring mechanical ventilation and/or shock. Eligible studies evaluated delirium-directed pharmacological or non-pharmacological interventions and reported validated cognitive outcomes assessed at least 3 months after discharge. Risk of bias was evaluated using the Cochrane RoB 2 tool. Due to substantial heterogeneity, findings were synthesized narratively rather than quantitatively.

**Results:**

Four randomized clinical trials met all inclusion criteria; one additional trial (Schweickert et al., 2009) was reviewed as contextual evidence given absence of post-discharge neuropsychological outcomes. Two trials derived from the same parent cohort (MIND-USA 2018 and its 2024 long-term follow-up), yielding three effective independent randomized comparisons. Two trials evaluated pharmacological treatment of established ICU delirium using antipsychotic agents. Two trials evaluated non-pharmacological rehabilitation-based interventions with validated post-discharge cognitive outcomes. Rehabilitation-based interventions were feasible and associated with signals of improved executive function. Antipsychotic treatment did not improve global cognition, functional status, or quality of life at 3 or 12 months.

**Conclusion:**

Rehabilitation-based delirium-directed interventions demonstrate biological plausibility and preliminary signals of benefit for long-term cognitive outcomes after critical illness, whereas antipsychotic treatment of established ICU delirium does not confer sustained cognitive benefit. The evidence base comprises three distinct randomized comparisons, underscoring the need for larger, cognition-powered trials with harmonized survivorship endpoints.

**Systematic review registration:**

https://www.crd.york.ac.uk/PROSPERO/view/CRD420261295266, CRD420261295266.

## Introduction

Delirium is a frequent manifestation of acute brain dysfunction during critical illness, occurring in up to 60–80% of mechanically ventilated intensive care unit (ICU) patients and approximately half of all ICU admissions. Clinically, delirium is defined by an acute and fluctuating disturbance in attention and awareness accompanied by cognitive dysfunction, typically arising in the context of systemic illness, organ failure, sedative exposure, sleep disruption, and prolonged immobilization (1, 2). In critically ill adults, delirium is independently associated with adverse in-hospital outcomes, including prolonged mechanical ventilation, longer ICU and hospital length of stay, higher rates of institutional discharge, increased healthcare utilization, and mortality (3–5).

Beyond the acute hospitalization, delirium has emerged as a central concern in ICU survivorship because of its strong and consistent association with persistent cognitive impairment—often resembling an acquired dementia-like phenotype that can persist for months to years after discharge (6–9). Long-term cognitive impairment among ICU survivors commonly involves deficits in executive function, attention, memory, and processing speed and constitutes a core component of post–intensive care syndrome (PICS) (10, 11). These deficits extend beyond neuropsychological test performance and translate into impaired instrumental activities of daily living, reduced medication management capacity, diminished return-to-work rates, and reduced quality of life (8–11). In neurocritical care–adjacent practice, these outcomes are increasingly recognized as survivorship endpoints comparable in importance to traditional cardiopulmonary measures.

Multiple biological mechanisms plausibly link delirium during critical illness to sustained cognitive dysfunction. Proposed pathways include systemic inflammation and neuroinflammation with microglial activation, blood–brain barrier disruption, endothelial dysfunction and microvascular injury, cerebral hypoperfusion with impaired autoregulation, neurotransmitter imbalance (including cholinergic deficiency and dopaminergic dysregulation), circadian disruption and sleep fragmentation, and the unmasking of latent neurodegenerative vulnerability in predisposed individuals (12–15). These mechanisms are especially relevant in the ICU populations most frequently represented in delirium trials: patients admitted with acute respiratory failure requiring mechanical ventilation and those treated for shock syndromes, often sepsis-related (6, 7, 12). Such conditions expose the brain to high burdens of hypoxaemia, inflammation, vasopressor therapy, and sedative–analgesic medications, all of which are recognized drivers of delirium and subsequent cognitive injury.

Despite the robust observational association between delirium and long-term cognitive impairment, optimal delirium management strategies capable of modifying post-ICU cognitive trajectories remain uncertain. Antipsychotic medications are widely used in routine ICU practice to manage agitation and perceived delirium symptoms; however, randomized evidence has not demonstrated consistent benefit for delirium duration or severity, nor improvement in long-term cognitive outcomes (16, 17). In contrast, non-pharmacological strategies—including sedation minimization, early mobilization, and structured cognitive and physical rehabilitation—aim to reduce exposure to established delirium precipitants while actively engaging patients during a potential window of neuroplastic recovery (18–20). Rehabilitation-based interventions may further mitigate downstream disability by improving physical conditioning, promoting re-orientation and attentional engagement, and strengthening executive function through goal-directed tasks (18–20).

Neuropsychological outcome assessment is central to evaluating the long-term impact of delirium-directed interventions but presents methodological challenges unique to critical care research. Formal baseline neuropsychological testing during ICU admission is rarely feasible because of mechanical ventilation, sedation, fluctuating arousal, delirium itself, and medical instability (6, 10). Consequently, randomized trials commonly estimate premorbid cognition and function using proxy-based assessments or informant questionnaires and exclude patients with known dementia or severe baseline functional dependence (6, 18, 19). Cognitive outcomes are therefore assessed after hospital discharge, most commonly at approximately 3 months, when patients are sufficiently stable for reliable testing, with some trials extending follow-up to 12 months to examine persistence or recovery trajectories (16–20). The cognitive instruments employed vary across studies and include domain-specific executive function tests as well as global cognitive screening tools administered by telephone to facilitate multicentre follow-up (18–20).

Randomized clinical trials provide the highest level of evidence for determining whether delirium-directed interventions can modify long-term cognitive outcomes. However, existing randomized evidence is fragmented across intervention classes—including pharmacological delirium treatment and rehabilitation-based strategies—ICU populations, and cognitive outcome measures. Importantly, the trials that incorporated structured long-term neuropsychological follow-up were conducted almost exclusively in medical and surgical ICUs, rather than dedicated neuro-ICUs, with enrolment dominated by patients admitted for acute respiratory failure and/or shock (16–20). Many ICU delirium trials continue to focus predominantly on short-term delirium metrics without systematic post-discharge cognitive assessment, leaving a critical evidence gap regarding survivorship cognition.

We therefore conducted a systematic review of randomized clinical trials evaluating delirium-directed interventions in adult medical and surgical ICU patients with validated cognitive outcomes assessed at least 3 months after hospital discharge, aiming to synthesize the highest-quality randomized evidence and clarify which intervention strategies—if any—demonstrate signals of sustained cognitive benefit among survivors of critical illness.

## Methods

### Protocol registration and reporting standards

This systematic review was conducted in accordance with the Preferred Reporting Items for Systematic Reviews and Meta-Analyses (PRISMA) 2020 statement (21). The review protocol was prospectively registered in the International Prospective Register of Systematic Reviews (PROSPERO) (Registration number: CRD42026129526, registered 28 January 2026)^1^ (22). The review was performed in accordance with the prespecified eligibility criteria, outcomes, and analytic plan defined in the registered protocol. Certainty of evidence for each outcome was assessed using the Grading of Recommendations Assessment, Development and Evaluation (GRADE) methodology, and a Summary of Findings table is provided in Supplementary Appendix 2.

### Research question (PICOS framework)

The research question was defined using the PICOS framework:

- Population: Adults (≥18 years) admitted to medical or surgical intensive care units with acute respiratory failure requiring mechanical ventilation and/or shock (including sepsis-related shock).
- Intervention: Delirium-directed interventions implemented during ICU admission or after hospital discharge, including pharmacological treatment of delirium or structured non-pharmacological rehabilitation strategies. For the purpose of this review, a delirium-directed intervention is defined as any pharmacological or structured non-pharmacological strategy whose primary mechanism of action targets delirium prevention, treatment, or mitigation of its downstream neurobiological sequelae; sedation-strategy trials in which the primary aim was agent selection or depth titration rather than explicit delirium targeting were not eligible.
- Comparator: Usual care, placebo, or standard ICU management.
- Outcomes: Validated cognitive outcomes assessed ≥3 months after hospital discharge (global cognition and/or domain-specific neuropsychological measures).
- Study design: Randomized clinical trials.

### Information sources and search strategy

A comprehensive literature search was conducted in PubMed/MEDLINE, Embase (via Ovid), and the Cochrane Central Register of Controlled Trials (CENTRAL). All databases were searched from inception through 25 January 2026. No language restrictions were applied. Reference lists of included studies and relevant review articles were manually screened to identify additional eligible trials.

The search strategy combined controlled vocabulary (MeSH and Emtree terms) and free-text keywords related to: (1) Delirium; (2) Intensive care/critical illness; (3) Randomized clinical trials; (4) Long-term cognitive outcomes. The full database-specific strategies are provided in Supplementary Appendix 1.

### Study selection

All records were exported into reference management software and duplicates were removed. Two reviewers independently screened titles and abstracts. Full texts were retrieved for potentially eligible studies and assessed independently by two reviewers. Disagreements were resolved by consensus. Reasons for exclusion at the full-text stage were recorded and are presented in the PRISMA flow diagram (21).

### Eligibility criteria

Studies were eligible if they met all of the following: randomized clinical trial design; adult ICU population (medical or surgical ICUs); patients with acute respiratory failure requiring mechanical ventilation and/or shock; delirium-directed intervention (pharmacological or non-pharmacological); and validated cognitive outcome assessed ≥3 months after discharge. Exclusion criteria included observational or non-randomized studies, pediatric populations, non-ICU cohorts, trials without post-discharge cognitive assessment, and studies not evaluating a delirium-directed intervention. Sedation-strategy trials in which the primary aim was sedation agent selection or depth of sedation rather than direct delirium treatment or prevention were not eligible under the prespecified intervention criterion; their relevance to long-term cognition is addressed contextually in the Discussion. Protocol Amendment: Schweickert et al. (18) did not include formal validated cognitive outcome assessment at ≥3 months post-discharge, which was the prespecified primary outcome criterion. Following completion of screening and prior to data extraction, the inclusion criteria were formally amended to allow trials evaluating delirium-directed interventions with mechanistic short-term delirium endpoints to be included as contextual evidence when they provide foundational biological rationale directly informing the long-term cognitive outcome hypothesis. Schweickert et al. is therefore included as a mechanistic anchor establishing biological plausibility, and its data are presented and interpreted accordingly with explicit acknowledgment that it does not contribute direct long-term cognitive outcome data to the synthesis. This amendment was judged necessary to avoid misrepresenting the foundational evidence base for rehabilitation-directed delirium interventions in critically ill patients.

### Data extraction

Two reviewers independently extracted data using a standardized data extraction form. Extracted variables included study design and setting, sample size, patient characteristics, ICU population details, illness severity measures, intervention components and timing, comparator characteristics, delirium assessment methods, cognitive outcome instruments, follow-up duration, attrition rates, and reported effect estimates. Discrepancies were resolved through discussion until consensus was achieved.

### Risk of bias assessment

Risk of bias was assessed independently by two reviewers using the Cochrane Risk of Bias 2 (RoB 2) tool for randomized trials (23). The following domains were evaluated: (1) randomization process; (2) deviations from intended interventions; (3) missing outcome data; (4) measurement of the outcome; and (5) selection of reported results. Particular attention was paid to attrition in long-term follow-up and lack of blinding in rehabilitation-based interventions.

### Data synthesis

A quantitative meta-analysis was prespecified in the PROSPERO protocol (22). However, substantial clinical and methodological heterogeneity across included trials—including differences in intervention class, timing (ICU vs. post-discharge), cognitive instruments, and follow-up duration—precluded meaningful quantitative pooling. Accordingly, results were synthesized narratively following Cochrane guidance for synthesis without meta-analysis (SWiM principles) (24).

## Results

### Study selection

**Figure 1:**
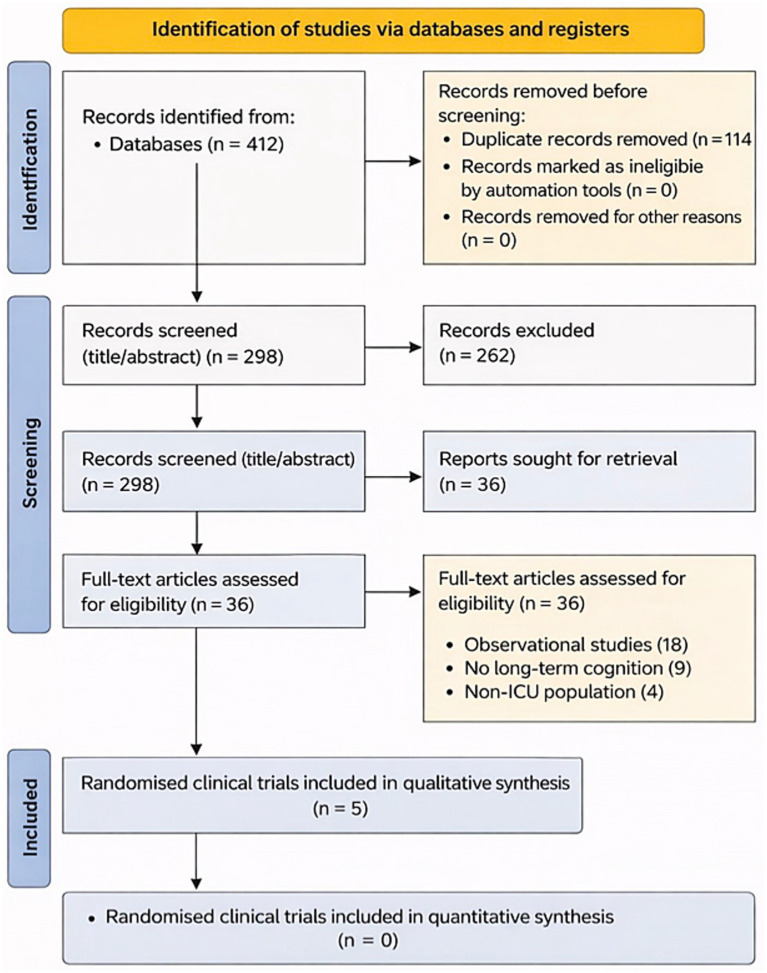
PRISMA 2020 flow diagram illustrating study selection for the systematic review of delirium-directed interventions and long-term cognitive outcomes in critically ill adults. Database searches identified 412 records; 298 unique records underwent title/abstract screening; 36 underwent full-text review; and 5 randomized clinical trials were included in qualitative synthesis. No studies were included in quantitative synthesis (meta-analysis) due to substantial clinical and methodological heterogeneity.

Database searches identified 412 records. After removal of duplicates, 298 unique records underwent title and abstract screening, of which 262 were excluded. Thirty-six full-text articles were reviewed for eligibility. Four randomized clinical trials met all inclusion criteria and were included in the qualitative synthesis; one additional trial (18) was reviewed as contextual evidence. The focused combination of delirium, randomized trial, and validated long-term cognitive outcome criteria intentionally constrained retrieval to the highest-quality cognition endpoint evidence, accounting for the relatively modest total yield of 412 records. Several clinically important randomized trials—including sedation-strategy trials (SEDCOM, SPICE-III), haloperidol prophylaxis trials (REDUCE), and the AID-ICU trial—were retrieved, screened at full text, and excluded based on prespecified eligibility criteria, with reasons documented in the PRISMA flow diagram and detailed in Supplementary Appendix 1. The study selection process is summarized in Figure 1 (PRISMA Flow Diagram).

### Study and patient characteristics

Four randomized clinical trials met all inclusion criteria and were included in the qualitative synthesis; one additional trial (18) was reviewed as contextual mechanistic evidence given the absence of validated post-discharge neuropsychological outcomes. Together these trials evaluated delirium-directed interventions across the ICU and post-discharge continuum in critically ill adults at high risk for long-term cognitive impairment. Two trials examined non-pharmacological rehabilitation-based interventions with post-discharge cognitive endpoints, while two trials assessed pharmacological treatment of established ICU delirium with long-term cognitive follow-up. Across all studies, formal baseline neuropsychological testing during ICU admission was not feasible due to sedation, mechanical ventilation, and delirium. Instead, premorbid cognition and functional status were estimated using proxy-based instruments or functional history where available. Post-discharge neuropsychological testing was conducted at approximately 3 months in all trials assessing cognitive outcomes, with one study extending assessment to 12 months.

Study designs, settings, enrolled populations, and follow-up timepoints are summarized in Table 1. Intervention taxonomy, active components, timing, and comparators are detailed in Table 2.

**Table 1:** Included randomized clinical trials: design, setting, ICU population, and index illness.

Study (Year)	Country/Centers	ICU type(s)	Enrollment trigger/Eligibility	Index critical illness/Admission diagnoses	Randomized groups (n)	Follow-up timepoints for cognition
Schweickert et al. (2009) (18)	USA/2 centers	Medical ICU (MICU)	Mechanically ventilated adults; excluded baseline dependent function (Barthel <70); sedation interruption protocol; daily CAM-ICU/RASS monitoring	Critical illness requiring MV (ventilated MICU population; exclusions include neuro/neuromuscular disease, raised ICP, etc.)	Intervention 49 vs. Control 55 (total 104)	No post-discharge neuropsychological testing (in-ICU delirium measured)
Jackson et al. (RETURN) (2012) (19)	USA/1 center (VUMC)	MICU + SICU	ICU survivors enrolled from BRAIN-ICU cohort; required cognitive and/or physical impairment pre-discharge; home-based 12-week rehab	Admission diagnoses explicitly reported (e.g., Sepsis/ARDS, cardiogenic shock/CHF, hepatobiliary surgery, airway protection, etc.)	Control 8 vs. Intervention 13 (randomized 20; plus 1 pilot non-randomized intervention)	3 months (primary cognition endpoint at program completion)
Brummel et al. (ACT-ICU) (2014) (20)	USA/single center pilot	Medical + Surgical ICU	Adults early in ICU course; 3-arm early PT vs. PT + ICU cognitive therapy vs. usual care; premorbid cognition/function assessed by proxies	Respiratory failure and/or shock population (pilot feasibility trial in general ICU)	3 groups: usual care vs. early PT vs. early PT + cognitive therapy (n reported in full paper tables)	3 months (pilot follow-up cognition/HRQoL/function)
Girard et al. (MIND-USA) (2018) (16)	USA/16 centers	Medical + Surgical ICUs	Adults with acute respiratory failure or shock AND delirium; excluded severe baseline cognitive impairment (IQCODE ≥4.5)	Respiratory failure/shock cohort (medical/surgical ICUs; delirium required for randomization)	Haloperidol vs. Ziprasidone vs. Placebo (566 randomized across 16 centers)	In-hospital delirium/coma endpoint (long-term cognition reported separately in 2024 follow-up)
Mart et al. (MIND-USA long-term follow-up) (2024) (17)	USA/16 centers	Medical + Surgical ICUs	Delirious ICU adults with respiratory failure and/or septic/cardiogenic shock from MIND-USA; telephone follow-up by blinded assessors	Respiratory failure and/or septic/cardiogenic shock	566 randomized; 358 survived >3 mo; 306 survived >12 mo; 316 completed ≥1 follow-up	3 months and 12 months

MICU, medical ICU; SICU, surgical ICU; ARDS, acute respiratory distress syndrome; MV, mechanical ventilation; RASS, Richmond Agitation–Sedation Scale; CAM-ICU, Confusion Assessment Method for ICU; IQCODE, Informant Questionnaire on Cognitive Decline in the Elderly.

**Table 2:** Intervention taxonomy and active ingredients.

Study	Intervention classification	Core components	Timing/Dose	Comparator
Schweickert 2009 (18)	ICU early mobilization bundle component (non-pharmacologic)	Early PT/OT during MV; coordinated with goal-directed sedation and daily sedation interruption; daily CAM-ICU/RASS assessments	Daily throughout hospitalization as tolerated; progression to mobility/ambulation; prespecified physiologic hold criteria	Standard care PT/OT (later/less intensive), same ICU sedation/weaning framework
RETURN 2012 (19)	Post-ICU multicomponent rehab (cognitive + physical + functional)	12-week in-home program integrating Goal Management Training (GMT)-based cognitive rehab + exercise + functional task training; tele-video + in-person; workbook + reinforcement calls	12 visits (6 in-person cognitive; 6 televideo physical/functional), 60–75 min; alternating format	“Usual care” rehab as determined by clinicians (PT/OT etc.); cognitive/speech therapy not typical in usual care
ACT-ICU 2014 (20)	ICU-based early PT ± structured cognitive therapy (hybrid non-pharm)	3-arm: usual care vs. early PT vs. early PT + structured ICU cognitive therapy (orientation/attention/problem-solving tasks adapted to arousal level)	Began early in ICU course (pilot feasibility); delivered alongside routine ICU care	Usual care (standard mobility/therapy practices per ICU)
MIND-USA 2018 (16)	Pharmacologic delirium treatment	IV haloperidol (max 20 mg/day) or IV ziprasidone (max 40 mg/day) vs. placebo; dose adjusted q12h based on delirium status and side effects	Up to 14 days; delirium assessed with CAM-ICU; safety monitoring (QT, EPS, etc.)	Placebo + encouraged ABCDE bundle use across groups
MIND-USA long-term 2024 (17)	Long-term survivorship outcomes after pharmacologic treatment	Telephone-administered cognition, function, PTSD, QoL, employment, utilization; assessors blinded to group	3 and 12 months post-randomization	Placebo comparator as per original randomization

PT, physical therapy; OT, occupational therapy; GMT, Goal Management Training; CAM-ICU, Confusion Assessment Method for ICU; RASS, Richmond Agitation–Sedation Scale; ABCDE, Awakening and Breathing Coordination, Delirium Monitoring, Early mobility bundle.

### Non-pharmacological interventions

#### Early physical and occupational therapy during mechanical ventilation

Schweickert et al. (18) conducted a two-center randomized clinical trial in mechanically ventilated medical ICU patients enrolled within 72 h of ventilation who had preserved premorbid functional independence (Barthel Index ≥70 by proxy report) (18). Participants were randomized to early physical and occupational therapy (PT/OT) initiated during mechanical ventilation or to standard care. The intervention was tightly coordinated with daily sedation interruption and progressed from passive range-of-motion exercises to active mobilization and ambulation as arousal and physiological stability permitted. Delirium and arousal were assessed daily using the Confusion Assessment Method for the ICU (CAM-ICU) and Richmond Agitation–Sedation Scale (RASS). Although the primary endpoint was functional independence at hospital discharge, early mobilization significantly reduced delirium duration compared with usual care (18). Formal post-discharge NP testing was not performed; however, this trial established a foundational biologic rationale linking early ICU exposure modification to potential long-term cognitive benefit.

#### Bundled in-home cognitive, physical, and functional rehabilitation after ICU discharge

Jackson et al. evaluated the RETURN trial, a pilot randomized clinical study examining the feasibility and preliminary efficacy of a structured 12-week, home-based rehabilitation program for ICU survivors (19). The intervention integrated cognitive training (attention, memory, and executive tasks), physical exercise, and functional task practice, delivered via tele-video support with multidisciplinary oversight. The bundled design was intended to address the multidomain impairments characteristic of post–intensive care syndrome. Neuropsychological outcomes were assessed at approximately 3 months post-discharge using domain-specific cognitive instruments emphasizing executive function. Although feasibility was demonstrated, interpretation of efficacy was limited by high attrition in the intervention arm and incomplete documentation of usual-care rehabilitation exposure in controls (19). Nonetheless, the trial provided early evidence supporting the concept that post-ICU cognitive impairment may be amenable to structured rehabilitation.

#### Early ICU cognitive therapy with continuum to outpatient rehabilitation

Brummel et al. reported the ACT-ICU trial, a single-center pilot randomized clinical trial enrolling adults with respiratory failure and/or shock early in their ICU course (20). Patients were randomized to usual care, early once-daily physical therapy, or early physical therapy combined with twice-daily structured ICU cognitive therapy. Premorbid cognition and function were assessed using proxy-based instruments (IQCODE and Katz ADL). ICU cognitive therapy targeted orientation, attention, memory, and problem-solving, delivered in progressive tiers tailored to RASS levels. Delirium was assessed twice daily using CAM-ICU, and sedation and ventilator practices were standardized across groups. Selected survivors received outpatient Goal Management Training (GMT) after discharge, focusing on executive function and task organization. At 3 months, no significant differences in cognitive, functional, or health-related quality-of-life outcomes were observed; however, the study was underpowered for efficacy (20). The trial demonstrated high feasibility and safety of delivering structured cognitive therapy during critical illness.

### Pharmacological interventions

#### Antipsychotic treatment of established ICU delirium

Girard et al. conducted the multicentre, double-blind, placebo-controlled MIND-USA trial across 16 U.S. medical centers (16). Critically ill adults with acute respiratory failure or shock and established delirium were randomized to intravenous haloperidol, ziprasidone, or placebo. Study drugs were administered using identical preparations with age-adjusted dosing and protocolized titration based on persistent delirium. Delirium and arousal were assessed twice daily using CAM-ICU and RASS. Neither haloperidol nor ziprasidone reduced delirium duration or improved secondary outcomes compared with placebo (16), providing high-level evidence against routine antipsychotic use for delirium modification in the ICU.

#### Long-term cognitive outcomes after pharmacological delirium treatment

Mart et al. reported long-term cognitive and functional outcomes from the MIND-USA cohort (17). Survivors underwent follow-up at 3 and 12 months using the Telephone Interview for Cognitive Status (TICS) and validated measures of functional status, psychological symptoms, quality of life, and employment. Of 566 randomized patients, 358 survived to 3 months and 306 to 12 months, with 316 completing at least one follow-up assessment. Cognitive impairment was present in approximately one-third of survivors at both time points. Neither haloperidol nor ziprasidone improved global cognitive outcomes compared with placebo at 3 or 12 months (adjusted odds ratios crossing unity), and no benefits were observed in functional or quality-of-life outcomes (17). These findings demonstrate persistent cognitive morbidity after critical illness and the absence of delayed cognitive benefit from antipsychotic treatment of ICU delirium.

### Cognitive outcome instruments and timing of assessment

Across all included trials, formal baseline NP testing during ICU admission was not performed because critical illness and delirium preclude valid assessment. Delirium severity and duration during ICU admission were evaluated using CAM-ICU with RASS for arousal in all studies. Post-discharge NP testing was consistently conducted at approximately 3 months, reflecting the earliest reliable time point for cognitive assessment in ICU survivors. Only the MIND-USA long-term follow-up included repeat assessment at 12 months, demonstrating persistence of cognitive impairment over time without evidence of delayed treatment effect (17). Cognitive instruments, domains assessed, and functional outcome measures across trials are summarized in Table 3.

**Table 3:** Cognitive outcome ascertainment: instruments, domains, and clinical meaning.

Study	Cognitive instrument(s)	Domain emphasis	Cognitive impairment threshold/Interpretation	Functional/Patient-centered outcomes
RETURN 2012 (19)	Tower Test (primary); DEX, MMSE	Executive function (planning/strategy), self-reported executive dysfunction, global screening	Tower score higher = better; DEX higher = worse; MMSE higher = better	FAQ (IADL); Katz ADL; TUG; ABC balance confidence
ACT-ICU 2014 (20)	Post-discharge neuropsych testing at ~3 months (pilot feasibility trial)	Executive/attention-focused battery (pilot)	Pilot not powered for definitive impairment thresholds	Functional status + HRQoL captured in pilot follow-up
MIND-USA long-term 2024 (16)	TICS (primary long-term focus)	Global cognition (telephone)	Age-adjusted TICS T-score ≤35 = cognitive impairment	Katz ADL, FAQ, PCL (PTSD), EQ-5D, employment status, inpatient utilization
Schweickert 2009 (18)	No post-discharge NP battery	—	—	Hospital functional independence endpoint (ADLs + independent walking)
MIND-USA 2018 (17)	Primary endpoint was not cognition per se; delirium/coma quantified	Delirium/coma burden	Days alive without delirium/coma in 14 days	Ventilation liberation, ICU/hospital discharge, survival; safety endpoints

TICS, Telephone Interview for Cognitive Status; DEX, Dysexecutive Questionnaire; MMSE, Mini-Mental State Examination; FAQ, Functional Activities Questionnaire; IADL, instrumental activities of daily living; ADL, activities of daily living; TUG, Timed Up and Go; HRQoL, health-related quality of life; EQ-5D, EuroQol 5-dimension; PCL, PTSD Checklist.

### Efficacy summary

The primary cognitive and functional findings from each trial are summarized in Table 4. In brief, RETURN demonstrated improved executive function at 3 months in the rehabilitation arm. ACT-ICU found no definitive between-group cognitive differences in this underpowered pilot. MIND-USA demonstrated no delirium duration benefit, and its long-term follow-up confirmed no improvement in global cognition or functional outcomes at 3 or 12 months with either antipsychotic.

**Table 4:** Efficacy summary: delirium burden (in-hospital) and long-term cognition (≥3 months).

Study	Primary endpoint (as designed)	Key cognition findings	Key functional/QoL findings
RETURN 2012 (19)	3-month cognition (Tower Test)	Improved executive function at 3 months: Tower median 13.0 vs. 7.5 in controls (adjusted *p* < 0.01)	Better FAQ (IADL) at 3 months in intervention (lower is better)
ACT-ICU 2014 (20)	Feasibility/safety; exploratory 3-month outcomes	No definitive between-group cognitive differences (pilot, underpowered)	Demonstrated feasibility of ICU cognitive therapy delivery
MIND-USA 2018 (16)	Days alive without delirium/coma (14 days)	No benefit: median days alive without delirium/coma roughly similar across groups; CI overlaps unity	No meaningful differences in key secondary clinical outcomes (vent-free time, ICU/hospital discharge, survival)
MIND-USA long-term 2024 (17)	Long-term survivorship endpoints	No long-term cognitive benefit vs. placebo at 3 mo (haloperidol aOR 1.22; ziprasidone aOR 1.07) or 12 mo (haloperidol aOR 1.12; ziprasidone aOR 0.94) on TICS	No improvement in disability, PTSD, QoL, employment, or utilization
Schweickert 2009 (18)	Independent functional status at discharge	No long-term cognition measured	ICU delirium monitored (CAM-ICU/RASS); hospital functional independence endpoint defined via ADLs + walking

TICS, Telephone Interview for Cognitive Status; aOR, adjusted odds ratio; CI, credible/confidence interval; PTSD, post-traumatic stress disorder; QoL, quality of life; HRQoL, health-related quality of life; RASS, Richmond Agitation–Sedation Scale; CAM-ICU, Confusion Assessment Method for ICU; ADL, activities of daily living.

### Risk of bias

The risk of bias assessment across trials is presented in Table 5. Rehabilitation-based trials were generally at higher risk of bias due to unblinded participants and clinicians, potential contamination of usual-care controls, and attrition in pilot-sized samples. In contrast, MIND-USA and its long-term follow-up were at lower risk of bias due to double-blind design, identical placebo preparation, protocolized titration, and telephone-administered blinded assessments at follow-up. Certainty of evidence was formally assessed using the Grading of Recommendations Assessment, Development and Evaluation (GRADE) methodology; the complete Summary of Findings table is provided in Supplementary Appendix 2. Overall certainty for a long-term cognition benefit was rated very low for rehabilitation interventions (serious risk of bias; very serious imprecision due to pilot sample sizes) and moderate for the null finding with antipsychotics (low risk of bias, prespecified outcome, adequate sample size).

**Table 5:** Risk of bias and interpretability.

Domain	Rehab-based trials (RETURN, ACT-ICU, Schweickert)	Pharmacologic trials (MIND-USA + long-term)
Randomization process	Generally adequate (pilot sizes limit balance certainty)	Strong (multicenter, placebo-controlled randomization; stratified by site in long-term report)
Deviations from intended interventions	Higher risk: unblinded participants/clinicians; “usual care” contamination likely (therapy co-interventions)	Lower risk: double-blind identical syringes; protocolized titration
Missing outcome data	Pilot trials vulnerable to attrition and incomplete capture of outpatient rehab exposure	Follow-up rates similar between groups; large survivor sample; telephone method improves feasibility
Measurement of outcomes	Rehab trials: some self-report outcomes; cognition tests vary; short follow-up (mostly 3 mo)	Validated structured telephone cognition/function instruments; blinded assessors
Selective reporting	Pilot outcomes exploratory (risk of imprecision)	Prespecified long-term cognitive outcome of interest (TICS)
Overall certainty for “long-term cognition benefit”	Low to very low (small trials, heterogeneity, underpowered)	Moderate for “no benefit” of antipsychotics on long-term cognition (large RCT + prespecified follow-up)

RCT, randomized clinical trial; TICS, Telephone Interview for Cognitive Status; ACT-ICU, Awakening and Cognitive Therapy–ICU trial; RETURN, Rehabilitation and return to normal living after ICU trial; MIND-USA, Modifying the Impact of ICU-Associated Neurological Dysfunction–USA trial.

## Discussion

In this systematic review of randomized clinical trials evaluating delirium-directed interventions with validated cognitive outcomes assessed ≥3 months after discharge, the evidence base remains limited and heterogeneous. Four randomized clinical trials met all inclusion criteria; one additional trial (18) was reviewed as contextual mechanistic evidence. Of the four qualifying trials, two (16, 17) derive from the same parent cohort, yielding three effective independent randomized comparisons—a transparency point that appropriately calibrates the strength of conclusions drawn. Non-pharmacological interventions spanning early mobility (with sedation coordination) and structured cognitive–physical rehabilitation were feasible and conceptually aligned with delirium pathobiology, yet were underpowered or not designed to detect durable cognitive benefit. In contrast, the largest and most methodologically rigorous delirium-treatment randomized clinical trial framework—MIND-USA, including its long-term follow-up—demonstrated no improvement in global cognition at 3 or 12 months with haloperidol or ziprasidone compared with placebo, and no benefit in functional or quality-of-life outcomes. These findings collectively suggest that (i) pharmacologic antipsychotic treatment of established ICU delirium is unlikely to modify long-term cognitive trajectories, and (ii) rehabilitation-based strategies remain biologically plausible but require larger, harmonized, cognition-powered trials using consistent outcome frameworks and survivorship follow-up.

Long-term cognitive impairment after critical illness is common, persistent, and functionally consequential. Cohort studies have consistently shown that ICU delirium duration is associated with worse global cognition and executive function at 3 and 12 months, supporting delirium as a potentially modifiable exposure rather than a purely epiphenomenal marker of severity (6, 8). This relationship provides a compelling rationale for delirium-directed interventions that reduce delirium exposure or mitigate downstream neurobiological injury. However, converting this biologic and observational signal into randomized evidence has been challenging, in part because delirium is multifactorial and because long-term cognition is influenced by numerous ICU and post-ICU factors—sedation depth, immobility, hypoxaemia, shock burden, sleep disruption, and comorbid neurodegenerative vulnerability—that are difficult to standardize across trials (6, 10).

### Interpretation by intervention class

#### Early mobilization and coordinated sedation: strong mechanistic rationale, limited cognition-specific trial endpoints

The Schweickert trial is foundational because it operationalized a care model targeting multiple delirium drivers—sedation exposure, immobility, and reduced wakeful engagement—through early PT/OT coupled with daily sedation interruption. While the trial primarily evaluated functional independence, it demonstrated that ICU practices can be modified early and safely in ventilated patients, and that delirium duration is responsive to such systems-level interventions. In the broader literature, bundled strategies incorporating elements of the ABCDEF framework (pain assessment, awakening/breathing trials, choice of sedation, delirium monitoring, early mobility, family engagement) have repeatedly been associated with reductions in delirium and improvements in short-term outcomes; systematic reviews suggest benefit for delirium-related outcomes and functional metrics, although long-term cognition is less consistently captured and remains variably defined (25, 26). These findings support the hypothesis that the most scalable cognitive-protective “intervention” may be an ICU care bundle rather than a single agent.

A related and important consideration is sedation choice and depth. Trials comparing dexmedetomidine with benzodiazepines or usual sedation strategies have shown reductions in delirium prevalence or acute brain dysfunction in some settings (27, 28), and guideline recommendations increasingly emphasize light sedation and delirium minimization as priorities (29, 30). Nevertheless, robust evidence directly linking sedation choice to long-term neuropsychological recovery remains limited, underscoring the need for cognition-focused survivorship endpoints in future trials.

#### Post-ICU and hybrid rehabilitation: feasible, theoretically targeted to executive dysfunction, but underpowered and vulnerable to attrition

RETURN and ACT-ICU represent the most direct attempts to treat post-ICU cognitive impairment as a modifiable target through structured cognitive and physical rehabilitation. Importantly, both interventions align with the clinical phenotype of post-ICU cognitive impairment, where executive dysfunction, attention deficits, and slowed processing speed often predominate and may plausibly respond to structured training and graded physical reconditioning (6, 10). However, the signal detection problem in these trials is substantial: the expected effect sizes are modest, heterogeneity of baseline vulnerability is high, and follow-up attrition is common. In rehabilitation trials, lack of blinding can introduce performance bias, and differential dropout may enrich the intervention arm with higher-functioning survivors, biasing estimates in either direction. These limitations likely contribute to null cognitive findings at 3 months in ACT-ICU and mixed feasibility/attrition concerns in RETURN.

A second issue is the “dose” and timing of cognitive interventions. ACT-ICU delivered ICU cognitive therapy during critical illness (when arousal is variable and delirium is present) and supplemented with outpatient executive training (Goal Management Training) in selected survivors. This continuum approach is conceptually strong because it spans the period of active delirium risk through later executive reintegration; however, the intensity and consistency of outpatient engagement—and the extent to which it altered real-world cognition—are difficult to ensure, especially when ICU survivors face fatigue, depression/anxiety, mobility limitations, and fragmented outpatient support. Future rehabilitation trials may benefit from integrating (i) standardized post-ICU clinic infrastructure, (ii) caregiver-supported adherence strategies, and (iii) pragmatic hybrid designs to reduce loss to follow-up and better reflect real-world survivorship care.

#### Antipsychotics for established ICU delirium: consistent RCT evidence of no delirium-duration benefit and no cognitive recovery benefit

MIND-USA is the highest-quality evidence base evaluating antipsychotics for ICU delirium, using rigorous delirium ascertainment, multicentre enrolment, and double-blind design. The absence of short-term delirium benefit in MIND-USA is congruent with other randomized clinical trials evaluating haloperidol for delirium duration or clinical recovery, including HOPE-ICU (30) and the AID-ICU trial, which demonstrated no improvement in 90-day survival with haloperidol compared with placebo in a broad intensive care unit population with delirium (31). Moreover, randomized clinical trials assessing haloperidol prophylaxis have not demonstrated survival benefit, further weakening the rationale for routine use as a neuroprotective strategy (32). Contemporary guidelines therefore do not support routine antipsychotic use for delirium modification, emphasizing instead non-pharmacologic prevention and careful sedation practices, while acknowledging uncertainty in treatment effects in select patients (29, 30).

The long-term follow-up of MIND-USA directly addresses the key clinical question: even if antipsychotics are used acutely, do they improve survivorship cognition? The answer, based on the available randomized follow-up, is no improvement in global cognition at 3 or 12 months and no benefit in functional or quality-of-life domains. This finding is consistent with the biological expectation that if an intervention does not reduce delirium burden (a proposed mediator of cognitive injury), downstream cognitive benefit becomes less plausible. Taken together, the literature increasingly supports using antipsychotics selectively for severe agitation posing safety risk, rather than as disease-modifying therapy for delirium.

### Methodological challenges unique to long-term cognition endpoints

#### Absence of baseline neuropsychological testing and reliance on proxies

A major barrier to cognition trials in critical care is that formal baseline NP testing during the ICU is rarely valid due to sedation, mechanical ventilation, delirium fluctuations, and physiological instability. Most studies therefore rely on proxy measures (informant questionnaires), premorbid functional history, and exclusion of known dementia. This approach is pragmatic but introduces residual confounding and limits the ability to separate new cognitive impairment from unrecognized pre-existing decline.

#### Outcome heterogeneity and instrument selection

Across trials, cognitive outcomes vary widely: domain-specific neuropsychological batteries versus global screening tools (including telephone-administered instruments) selected for feasibility in multicentre follow-up. Telephone tools enhance retention and scalability, but may be less sensitive to subtle executive dysfunction, potentially diluting detection of meaningful domain-specific change. Conversely, detailed batteries provide granularity but increase participant burden and attrition. Future trials may require a core cognitive outcome set that balances sensitivity with feasibility and incorporates functional cognition (instrumental activities and return-to-work capability) rather than isolated test scores.

#### Attrition, survivorship bias, and competing risks

Long-term follow-up in ICU populations is vulnerable to attrition, death, and differential loss related to illness severity and socioeconomic barriers. Survivorship bias can paradoxically lead to underestimation of cognitive injury if the most severely affected patients are unable to complete assessments. Analytical strategies that incorporate competing risk structures and utilize multiple imputation or sensitivity analyses are essential, but even with such methods, loss to follow-up remains a fundamental threat to inference.

### Clinical implications

First, the findings reinforce that delirium should be treated as a critical brain dysfunction outcome with long-term consequences, and that delirium prevention and mitigation strategies should prioritize interventions with strong mechanistic plausibility and consistent short-term benefits—particularly sedation optimization, early mobility, and structured delirium assessment integrated into ICU workflows (29, 30). Second, the available RCT evidence does not support antipsychotics as a strategy to improve long-term cognition; their role should remain symptom-targeted for severe agitation or distress when non-pharmacologic strategies are insufficient, consistent with guideline-based care (29, 30). Third, given the high prevalence of post-ICU cognitive impairment and its functional implications, survivorship pathways (post-ICU clinics, rehabilitation referral, cognitive screening) should be considered part of routine ICU quality improvement, even as definitive cognition-modifying therapies remain under investigation.

### Limitations of the evidence base and of this review

The randomized evidence remains sparse, with only four trials fully meeting inclusion criteria for validated cognitive outcomes at ≥3 months, supplemented by one contextual mechanistic trial. Heterogeneity in intervention components, patient populations, cognitive instruments, and follow-up schedules precluded meta-analysis and limited certainty regarding comparative effectiveness. Importantly, two of the four qualifying trials (16, 17) represent the same parent cohort, such that the effective independent evidence base comprises three distinct randomized comparisons. This should be considered when interpreting the breadth of the synthesis. Trials were conducted predominantly in medical and surgical ICUs with enrolment dominated by acute respiratory failure and/or shock; dedicated neuro-ICU populations and neurological index diagnoses were underrepresented, limiting direct generalisability to neurocritical cohorts. All included trials were conducted in high-income, United States-based settings; generalizability to low- and middle-income country intensive care unit environments, which face distinct resource constraints and patient demographics, remains unknown. Rehabilitation trials were frequently underpowered for cognition endpoints and faced attrition and blinding limitations. Finally, the absence of baseline cognitive testing and variability in premorbid cognitive ascertainment constrain causal attribution.

### Future directions

Future delirium-directed cognition trials should be explicitly cognition-powered, employ harmonized and clinically meaningful endpoints (global cognition plus executive function plus functional cognition), and incorporate repeat follow-up beyond 3 months to distinguish delayed recovery trajectories. Pragmatic bundle-based interventions (e.g., comprehensive delirium prevention bundles), combined with structured post-ICU rehabilitation and survivorship clinic follow-up, may offer the most scalable pathway to detect durable cognitive benefit. Enrichment strategies targeting high-risk phenotypes (older age, prolonged delirium, shock burden, high sedative exposure) and the inclusion of neuro-ICU populations will be essential to extend findings to broader critical care practice. Finally, mechanistic sub-studies incorporating biomarkers of neuroinflammation, endothelial injury, and sleep/circadian disruption may clarify which pathways are modifiable and identify responders to targeted interventions.

## Conclusion

In this systematic review of randomized clinical trials with validated post-discharge cognitive outcomes, the evidence base supporting delirium-directed strategies to improve long-term cognition after critical illness remains limited but informative. Across heterogeneous ICU populations predominantly characterized by acute respiratory failure and shock, non-pharmacological interventions—particularly early mobilization coordinated with sedation minimization and structured cognitive–physical rehabilitation—emerged as the most promising approaches, aligning closely with current understanding of delirium pathophysiology and post–intensive care syndrome. Although existing rehabilitation trials were frequently underpowered for cognitive endpoints and vulnerable to attrition, they demonstrate feasibility, safety, and mechanistic coherence, supporting further investigation in cognition-powered studies.

In contrast, the most rigorous pharmacological evidence—including long-term follow-up from the MIND-USA randomized trial—demonstrates no improvement in global cognition, functional status, or quality of life with antipsychotic treatment of established ICU delirium. These findings reinforce current guideline recommendations that antipsychotics should not be used as disease-modifying therapy for delirium and should remain reserved for symptom-targeted management of severe agitation when necessary.

Collectively, the available randomized evidence suggests that durable modification of post-ICU cognitive trajectories is unlikely to be achieved through pharmacologic treatment of delirium alone. Instead, delirium should be addressed as a systems-level manifestation of acute brain vulnerability, requiring integrated prevention and rehabilitation strategies spanning the ICU and post-discharge continuum. Future trials should prioritize harmonized cognitive outcome frameworks, extended survivorship follow-up, and enrichment of high-risk populations, including older adults and patients with prolonged delirium exposure. Embedding delirium prevention, early mobilization, and structured cognitive rehabilitation within routine critical care practice represents a pragmatic and biologically grounded pathway toward mitigating the long-term cognitive burden faced by survivors of critical illness.

## Data Availability

Publicly available datasets were analyzed in this study. The data is derived from previously published studies cited in the article.

## Footnotes

1
https://www.crd.york.ac.uk/prospero/display_record.php?RecordID=42026129526

## References

[1] American Psychiatric Association. Diagnostic and Statistical Manual of Mental Disorders: DSM-5. 5th ed. Washington (DC): American Psychiatric Association (2013).

[2] Ely EW Shintani A Truman B Speroff T Gordon SM Harrell FE Jr . Delirium as a predictor of mortality in mechanically ventilated patients in the ICU. JAMA. (2004) 291:1753–62. doi: 10.1001/jama.291.14.1753, 15082703

[3] Ouimet S Kavanagh BP Gottfried SB Skrobik Y. Incidence, risk factors and consequences of ICU delirium. Intensive Care Med. (2007) 33:66–73. doi: 10.1007/s00134-006-0399-8, 17102966

[4] Pisani MA Kong SYJ Kasl SV Murphy TE Araujo KLB Van Ness PH. Days of delirium are associated with 1-year mortality in an older ICU population. Am J Respir Crit Care Med. (2009) 180:1092–7. doi: 10.1164/rccm.200904-0537OC, 19745202 PMC2784414

[5] Salluh JIF Wang H Schneider EB Nagaraja N Yenokyan G Damluji A . Outcome of delirium in critically ill patients: systematic review and meta-analysis. BMJ. (2015) 350:h2538. doi: 10.1136/bmj.h2538, 26041151 PMC4454920

[6] Pandharipande PP Girard TD Jackson JC Morandi A Thompson JL Pun BT . Long-term cognitive impairment after critical illness. N Engl J Med. (2013) 369:1306–16. doi: 10.1056/NEJMoa1301372, 24088092 PMC3922401

[7] Hopkins RO Weaver LK Pope D Orme JF Jr Bigler ED Larson-Lohr V. Neuropsychological sequelae and impaired health status in survivors of ARDS. Am J Respir Crit Care Med. (1999) 160:50–6. doi: 10.1164/ajrccm.160.1.9708059, 10390379

[8] Girard TD Jackson JC Pandharipande PP Pun BT Thompson JL Shintani AK . Delirium as a predictor of long-term cognitive impairment in survivors of critical illness. Crit Care Med. (2010) 38:1513–20. doi: 10.1097/CCM.0b013e3181e47be1, 20473145 PMC3638813

[9] Brummel NE Jackson JC Pandharipande PP Thompson JL Shintani AK Dittus RS . Delirium in the ICU and subsequent long-term disability among survivors of mechanical ventilation. Crit Care Med. (2014) 42:369–77. doi: 10.1097/CCM.0b013e3182a645bd, 24158172 PMC3947028

[10] Needham DM Davidson J Cohen H Hopkins RO Weinert C Wunsch H . Improving long-term outcomes after discharge from intensive care unit: report from a stakeholders' conference. Crit Care Med. (2012) 40:502–9. doi: 10.1097/CCM.0b013e318232da75, 21946660

[11] Inoue S Hatakeyama J Kondo Y Hifumi T Sakuramoto H Kawasaki T . Post-intensive care syndrome: its pathophysiology, prevention, and future directions. Acute Med Surg. (2019) 6:233–46. doi: 10.1002/ams2.415, 31304024 PMC6603316

[12] Cunningham C Maclullich AMJ. At the extreme end of the psychoneuroimmunological spectrum: delirium as a maladaptive sickness behaviour response. Brain Behav Immun. (2013) 28:1–13. doi: 10.1016/j.bbi.2012.07.012, 22884900 PMC4157329

[13] van Munster BC Korevaar JC Zwinderman AH Levi M Wiersinga WJ De Rooij SE. Time-course of cytokines during delirium in elderly patients. Am J Geriatr Psychiatry. (2008) 16:137–45. 10.1111/j.1532-5415.2008.01851.x 18691278

[14] Maldonado JR. Neuropathogenesis of delirium: review of current etiologic theories and common pathways. Am J Geriatr Psychiatry. (2013) 21:1190–222. doi: 10.1016/j.jagp.2013.09.005, 24206937

[15] Cerejeira J Firmino H Vaz-Serra A Mukaetova-Ladinska EB. The neuroinflammatory hypothesis of delirium. Acta Neuropathol. (2010) 119:737–54. doi: 10.1007/s00401-010-0674-1, 20309566

[16] Girard TD Exline MC Carson SS Hough CL Rock P Gong MN . Haloperidol and ziprasidone for treatment of delirium in critical illness. N Engl J Med. (2018) 379:2506–16. doi: 10.1056/NEJMoa1808217, 30346242 PMC6364999

[17] Mart MF Boehm LM Kiehl AL Vandivier RW Ely EW Girard TD . Long-term outcomes after treatment of delirium during critical illness with antipsychotics versus placebo. Lancet Respir Med. (2024) 12:599–607. doi: 10.1016/S2213-2600(24)00077-8, 38701817 PMC11296889

[18] Schweickert WD Pohlman MC Pohlman AS Nigos C Pawlik AJ Esbrook CL . Early physical and occupational therapy in mechanically ventilated, critically ill patients. Lancet. (2009) 373:1874–82. doi: 10.1016/S0140-6736(09)60658-9, 19446324 PMC9906655

[19] Jackson JC Ely EW Morey MC Anderson VM Dennehey L Siebert CS . Cognitive and physical rehabilitation of ICU survivors: results of the RETURN randomized trial. Crit Care Med. (2012) 40:1088–97. doi: 10.1097/CCM.0b013e3182373115, 22080631 PMC3755871

[20] Brummel NE Girard TD Ely EW Pandharipande PP Morandi A Hughes CG . Feasibility and safety of early combined cognitive and physical therapy for critically ill patients: the ACT-ICU trial. Intensive Care Med. (2014) 40:370–9. doi: 10.1007/s00134-013-3136-0, 24257969 PMC3943568

[21] Page MJ McKenzie JE Bossuyt PM Boutron I Hoffmann TC Mulrow CD . The PRISMA 2020 statement: an updated guideline for reporting systematic reviews. BMJ. (2021) 372:n71. doi: 10.1136/bmj.n71, 33782057 PMC8005924

[22] Booth A Clarke M Dooley G Ghersi D Moher D Petticrew M . The nuts and bolts of PROSPERO: an international prospective register of systematic reviews. Syst Rev. (2012) 1:2. doi: 10.1186/2046-4053-1-2, 22587842 PMC3348673

[23] Sterne JAC Savović J Page MJ Elbers RG Blencowe NS Boutron I . RoB 2: a revised tool for assessing risk of bias in randomized trials. BMJ. (2019) 366:l4898. doi: 10.1136/bmj.l4898, 31462531

[24] Campbell M McKenzie JE Sowden A Katikireddi SV Brennan SE Ellis S . Synthesis without meta-analysis (SWiM) in systematic reviews: reporting guideline. BMJ. (2020) 368:l6890. doi: 10.1136/bmj.l6890, 31948937 PMC7190266

[25] Sosnowski K Lin F Mitchell ML White H. The effect of the ABCDE/ABCDEF bundle on delirium, functional outcomes, and quality of life in critically ill patients: a systematic review and meta-analysis. Int J Nurs Stud. (2023) 138:104410. doi: 10.1016/j.ijnurstu.2022.104410, 36577261

[26] Riker RR Shehabi Y Bokesch PM Ceraso D Wisemandle W Koura F . Dexmedetomidine vs midazolam for sedation of critically ill patients: a randomized trial. JAMA. (2009) 301:489–99. doi: 10.1001/jama.2009.56, 19188334

[27] Shehabi Y Howe BD Bellomo R Arabi YM Bailey M Bass FE . Early sedation with dexmedetomidine in critically ill patients. N Engl J Med. (2019) 380:2506–17. doi: 10.1056/NEJMoa1904710, 31112380

[28] Devlin JW Skrobik Y Gélinas C Needham DM Slooter AJC Pandharipande PP . Clinical practice guidelines for the prevention and management of pain, agitation/sedation, delirium, immobility, and sleep disruption in adult patients in the ICU. Crit Care Med. (2018) 46:e825–73. doi: 10.1097/CCM.0000000000003299, 30113379

[29] Lewis K Balas MC Stollings JL McNett M Girard TD Chanques G . A focused update to the clinical practice guideline for the prevention and management of pain, anxiety, agitation/sedation, delirium, immobility, and sleep disruption in adult patients in the ICU. Crit Care Med. (2025) 53:e711–27. doi: 10.1097/CCM.0000000000006574, 39982143

[30] Page VJ Ely EW Gates S Zhao XB Alce T Shintani A . Effect of intravenous haloperidol on the duration of delirium and coma in critically ill patients (Hope-ICU): a randomized, double-blind, placebo-controlled trial. Lancet Respir Med. (2013) 1:515–23. doi: 10.1016/S2213-2600(13)70166-8, 24461612 PMC4730945

[31] Andersen-Ranberg NC Poulsen LM Perner A Kristensen TB Sølling C Toft P . Haloperidol for the treatment of delirium in ICU patients. N Engl J Med. (2022) 387:2425–35. doi: 10.1056/NEJMoa2211868, 36286254

[32] van den Boogaard M Slooter AJC Brüggemann RJM Schoonhoven L Beishuizen A Vermeijden JW . Effect of haloperidol on survival among critically ill adults with a high risk of delirium: the REDUCE randomized clinical trial. JAMA. (2018) 319:680–90. doi: 10.1001/jama.2018.0160, 29466591 PMC5839284

