# Supplementary material for: Delirium-directed interventions and long-term cognitive outcomes in critically ill adults: a systematic review of randomized clinical trials
Source: Front Neurol. 2026 May 29;17:1833028. doi: 10.3389/fneur.2026.1833028 (PMC13262196; doi:10.3389/fneur.2026.1833028)
Supplement: Supplementary file 1 [file Supplementary_file_1.pdf]

Supplementary Appendix 1: Full Database Search Strategies

Review title: Delirium-Directed Interventions and Long-Term Cognitive Outcomes in Critically Ill Adults: A Systematic Review of Randomized Clinical Trials

PROSPERO Registration: CRD42026129526

Date of searches: Inception to 25 January 2026

Databases searched: PubMed/MEDLINE; Embase (via Ovid); Cochrane Central Register of Controlled Trials (CENTRAL)

Note: No language restrictions were applied. Reference lists of included studies and relevant systematic reviews were manually screened for additional eligible trials. The search strategy followed PRISMA-S (Preferred Reporting Items for Systematic Reviews and Meta-Analyses — Literature Search extension) reporting guidelines.

1. PubMed/MEDLINE Search Strategy

Database: PubMed/MEDLINE. Platform: National Library of Medicine. Date searched: 25 January 2026. Records retrieved: 187.

```
#1  "Delirium"[MeSH Terms]
#2  delirium[tiab] OR "acute brain dysfunction"[tiab] OR "acute confusional state"[tiab]
#3  #1 OR #2
#4  "Intensive Care Units"[MeSH Terms] OR "Critical Care"[MeSH Terms] OR "Critical Illness"[MeSH Terms]
#5  "intensive care unit"[tiab] OR ICU[tiab] OR "critical care"[tiab] OR "critical illness"[tiab] OR "critically ill"[tiab] OR "mechanically ventilated"[tiab] OR "mechanical ventilation"[tiab] OR "respiratory failure"[tiab] OR sepsis[tiab] OR shock[tiab]
#6  #4 OR #5
#7  "Randomized Controlled Trial"[pt] OR "Randomized Controlled Trials as Topic"[MeSH Terms]
#8  randomized[tiab] OR randomised[tiab] OR RCT[tiab] OR "clinical trial"[tiab] OR "controlled trial"[tiab]
#9  #7 OR #8
#10  "Cognition Disorders"[MeSH Terms] OR "Cognitive Dysfunction"[MeSH Terms] OR "Neuropsychological Tests"[MeSH Terms] OR "Cognition"[MeSH Terms]
#11  "cognitive outcome"[tiab] OR "cognitive impairment"[tiab] OR "cognitive function"[tiab] OR "neuropsychological"[tiab] OR "long-term cognitive"[tiab] OR "cognitive follow-up"[tiab] OR "post-intensive care syndrome"[tiab] OR PICS[tiab] OR "post-ICU"[tiab] OR "survivorship"[tiab] OR "long-term outcome"[tiab]
#12  #10 OR #11
#13  #3 AND #6 AND #9 AND #12
Limits applied: Humans; Date: inception to 25 January 2026; No language restriction
```

2. Embase (via Ovid) Search Strategy

Database: Embase. Platform: Ovid. Date searched: 25 January 2026. Records retrieved: 163.

```
#1  exp delirium/ OR exp acute confusional state/
#2  (delirium OR "acute brain dysfunction" OR "acute confusional state").ti,ab.
#3  #1 OR #2
#4  exp intensive care unit/ OR exp critical illness/ OR exp mechanical ventilation/ OR exp respiratory failure/ OR exp sepsis/ OR exp shock/
#5  ("intensive care unit" OR ICU OR "critical care" OR "critically ill" OR "mechanically ventilated" OR "mechanical ventilation" OR "respiratory failure" OR sepsis OR shock).ti,ab.
#6  #4 OR #5
```

```
#7  exp randomized controlled trial/ OR exp clinical trial/
#8  (randomized OR randomised OR RCT OR "clinical trial" OR "controlled
trial").ti,ab.
#9  #7 OR #8
#10  exp cognition disorder/ OR exp neuropsychological test/ OR exp long term
outcome/
#11  ("cognitive outcome" OR "cognitive impairment" OR "cognitive function" OR
neuropsychological OR "long-term cognitive" OR "post-intensive care syndrome"
OR PICS OR "post-ICU" OR survivorship OR "long-term outcome").ti,ab.
#12  #10 OR #11
#13  #3 AND #6 AND #9 AND #12
Limits applied: Humans; Date: inception to 25 January 2026; No language
restriction
```

3. Cochrane Central Register of Controlled Trials (CENTRAL) Search Strategy

Database: CENTRAL (Cochrane Library). Platform: Wiley. Date searched: 25 January 2026. Records retrieved: 62.

```
#1  MeSH descriptor: [Delirium] explode all trees
#2  delirium:ti,ab OR "acute brain dysfunction":ti,ab OR "acute confusional
state":ti,ab
#3  #1 OR #2
#4  MeSH descriptor: [Intensive Care Units] explode all trees OR MeSH
descriptor: [Critical Care] explode all trees OR MeSH descriptor: [Critical
Illness] explode all trees
#5  "intensive care unit":ti,ab OR ICU:ti,ab OR "critical care":ti,ab OR
"critically ill":ti,ab OR "mechanically ventilated":ti,ab OR "respiratory
failure":ti,ab OR sepsis:ti,ab OR shock:ti,ab
#6  #4 OR #5
#7  MeSH descriptor: [Cognition Disorders] explode all trees OR MeSH
descriptor: [Neuropsychological Tests] explode all trees
#8  "cognitive outcome":ti,ab OR "cognitive impairment":ti,ab OR "cognitive
function":ti,ab OR neuropsychological:ti,ab OR "long-term cognitive":ti,ab OR
"post-intensive care syndrome":ti,ab OR PICS:ti,ab OR "long-term outcome":ti,ab
#9  #7 OR #8
#10  #3 AND #6 AND #9
Filter: Trials only; Date: inception to 25 January 2026
```

4. Total Records and Deduplication

Total records retrieved across all databases: 412.

After automated and manual deduplication: 298 unique records proceeded to title and abstract screening.

Duplicate removal was performed in Rayyan systematic review software and verified manually.

5. Key Trials Screened and Excluded at Full-Text Stage (Selected Examples)

The following trials were identified as potentially relevant and retrieved for full-text review but were excluded at the eligibility assessment stage. Reasons for exclusion are documented below and correspond to the PRISMA flow diagram (Figure 1).

- Riker RR, et al. (JAMA 2009) — Dexmedetomidine vs midazolam (SEDCOM): Primary aim was sedation strategy, not a delirium-directed intervention; no ≥3-month validated cognitive outcome. Excluded per intervention criterion.
- Shehabi Y, et al. (NEJM 2019) — SPICE-III: Primary aim was early versus late sedation commencement; no ≥3-month validated cognitive outcome. Excluded per intervention criterion.
- Page VJ, et al. (Lancet Respir Med 2013) — HOPE-ICU: Haloperidol prophylaxis trial; no ≥3-month validated neuropsychological outcome reported. Excluded per outcome criterion.

- van den Boogaard M, et al. (JAMA 2018) — REDUCE: Haloperidol prophylaxis; primary outcome was 28-day survival; no ≥3-month validated cognitive assessment. Excluded per outcome criterion.
- Morandi A, et al. — MENDS (Pandharipande et al., NEJM 2007): Dexmedetomidine vs lorazepam sedation strategy trial; primary outcome was delirium/coma-free days; no ≥3-month validated cognitive endpoint. Excluded per intervention and outcome criteria.
- Pandharipande P, et al. — MENDS-2 (NEJM 2022, Hughes et al.): Dexmedetomidine vs propofol sedation; primary outcome was delirium/coma-free days; no ≥3-month validated cognitive endpoint. Excluded per intervention and outcome criteria.
- Andersen-Ranberg NC, et al. (NEJM 2022) — AID-ICU: Haloperidol vs placebo in ICU patients with delirium; primary outcome was 90-day survival; no ≥3-month validated neuropsychological cognitive assessment reported. Excluded per outcome criterion. Cited in Discussion as supportive evidence for the null effect of antipsychotics.

This list is illustrative of the most clinically relevant exclusions and is not exhaustive. Complete exclusion reasons for all full-text articles reviewed are available from the corresponding author upon request.
