# Supplementary material for: Delirium-directed interventions and long-term cognitive outcomes in critically ill adults: a systematic review of randomized clinical trials
Source: Front Neurol. 2026 May 29;17:1833028. doi: 10.3389/fneur.2026.1833028 (PMC13262196; doi:10.3389/fneur.2026.1833028)
Supplement: Supplementary file 2 [file Supplementary_file_2.docx]

# **Supplementary Appendix 2: GRADE Summary of Findings Table**

Delirium-Directed Interventions and Long-Term Cognitive Outcomes in Critically Ill Adults

Population: Adult ICU survivors (acute respiratory failure and/or shock). Intervention: Delirium-directed pharmacological or non-pharmacological rehabilitation interventions. Comparator: Usual care or placebo. Setting: Medical and surgical intensive care units, United States. Certainty assessed using GRADE methodology (Grading of Recommendations Assessment, Development and Evaluation). Certainty symbols: ⊕⊕⊕⊕ High; ⊕⊕⊕◯ Moderate; ⊕⊕◯◯ Low; ⊕◯◯◯ Very Low.

| **Outcome** | **No. of Studies** | **No. of Participants** | **Effect Estimate / Key Findings** | **Certainty of Evidence (GRADE)** | **Importance** |
| --- | --- | --- | --- | --- | --- |
| **Non-Pharmacological Rehabilitation-Based Interventions** | | | | | |
| Long-term cognitive outcomes (≥3 months) — Rehabilitation-based interventions (RETURN, ACT-ICU) | 2 RCTs | ~33 (RETURN pilot); ~60 (ACT-ICU pilot) | RETURN: Tower Test median 13.0 vs 7.5 (intervention vs control, p<0.01 for executive function). ACT-ICU: No significant between-group cognitive differences (pilot, underpowered). | ⊕◯◯◯ Very Low (serious risk of bias — unblinded; very serious imprecision — pilot sample sizes; inconsistency — directionally divergent results across trials) | Critical |
| Functional outcomes (≥3 months) — Rehabilitation-based interventions | 2 RCTs | ~33–60 | RETURN: Improved FAQ (functional activities) scores. ACT-ICU: No definitive functional differences in pilot. | ⊕◯◯◯ Very Low (serious risk of bias; very serious imprecision) | Important |
| **Pharmacological Interventions (Antipsychotics for Established ICU Delirium)** | | | | | |
| Long-term global cognition (3 months) — Antipsychotics (haloperidol or ziprasidone) vs placebo (MIND-USA long-term follow-up) | 1 RCT (parent + long-term follow-up cohort) | 316 with ≥1 follow-up (358 alive at 3 months) | No benefit: haloperidol aOR 1.22 (95% CI 0.61–2.46); ziprasidone aOR 1.07 (95% CI 0.52–2.22). ~33% of survivors had cognitive impairment regardless of treatment. | ⊕⊕⊕◯ Moderate (downgraded one level for imprecision in subgroups; otherwise low risk of bias, blinded, prespecified outcome) | Critical |
| Long-term global cognition (12 months) — Antipsychotics vs placebo (MIND-USA long-term follow-up) | 1 RCT | 306 alive at 12 months | No benefit: haloperidol aOR 1.12 (95% CI 0.55–2.30); ziprasidone aOR 0.94 (95% CI 0.45–1.97). | ⊕⊕⊕◯ Moderate (same as above; some attrition at 12 months) | Critical |
| Functional status and quality of life (3 and 12 months) — Antipsychotics vs placebo | 1 RCT | 316 with ≥1 follow-up | No significant differences in Katz ADL, FAQ, EQ-5D, PTSD (PCL), employment status, or healthcare utilization between groups. | ⊕⊕⊕◯ Moderate | Important |

Abbreviations: RCT, randomized clinical trial; aOR, adjusted odds ratio; CI, confidence interval; TICS, Telephone Interview for Cognitive Status; FAQ, Functional Activities Questionnaire; ADL, activities of daily living; EQ-5D, EuroQol 5-Dimension; PCL, PTSD Checklist; PTSD, post-traumatic stress disorder; ICU, intensive care unit; GRADE, Grading of Recommendations Assessment, Development and Evaluation.
